# The Psychological Implications of Automated Insulin Delivery Systems in Type 1 Diabetes Care

**DOI:** 10.3389/fcdhc.2022.846162

**Published:** 2022-05-03

**Authors:** Giesje Nefs

**Affiliations:** ^1^Department of Medical Psychology, Radboud University Medical Center, Radboud Institute for Health Sciences, Nijmegen, Netherlands; ^2^Diabeter, National Treatment and Research Center for Children, Adolescents and Adults With Type 1 Diabetes, Rotterdam, Netherlands; ^3^Department of Medical and Clinical Psychology, Center of Research On Psychological Disorders and Somatic Diseases (CoRPS), Tilburg University, Tilburg, Netherlands

**Keywords:** diabetes, technology, closed-loop, self-management, behavior, psychology

## Abstract

Automated insulin delivery (AID) systems have brought important glycemic benefits to type 1 diabetes management. The present paper provides an overview of their psychological implications. Trials and real-world observational studies report improvements in diabetes-specific quality of life, with qualitative work describing reduced management burden, increased flexibility and improved relationships. Not all experiences are positive, however, evidenced by dropping algorithm use soon after device initiation. Apart from finance and logistics, reasons for discontinuation include technology frustrations, wear-related issues and unmet glycemic and work load expectations. New challenges include distrust in proper AID functioning, overreliance and deskilling, compensatory behaviors to override or trick the system and optimize time in range, and concerns related to wearing multiple devices on the body. Research efforts may focus on incorporating a diversity perspective, updating existing person-reported outcome measures according to new technology developments, addressing implicit or explicit health professional bias in technology access, examining the merits of incorporating stress reactivity in the AID algorithm, and developing concrete approaches for psychological counseling and support related to technology use. An open dialogue with health professionals and peers about expectations, preferences and needs may foster the collaboration between the person with diabetes and the AID system.

## Introduction

Recent years have witnessed rapid advancements in technological devices assisting in insulin delivery and glucose monitoring in type 1 diabetes (T1D) care, with the goal of improving glucose levels to more closely resemble those in people without diabetes. Integration of insulin pump and sensor technologies has progressed from low-glucose and predictive low-glucose suspension (insulin cessation when sensor glucose crosses or is predicted to cross the low threshold level) to automated insulin delivery (AID) systems (1–3).

Also called artificial pancreas or hybrid closed-loop, these systems focus on algorithm-driven partially automated insulin delivery based on sensor readings. Initial hybrid systems combining automated basal insulin delivery with manual meal and residual correction boluses have swiftly been advanced with autocorrection boluses, meal detection, as well as more personalized treatment options (e.g. multiple target set points) and new algorithm enhancements are underway (1–3).

There are several commercial AID systems available in routine diabetes care, with multiple others being developed [for an overview, please refer to (1, 3)]. Dual-hormone closed loop systems are also finding their way to the market, adding other hormones to more closely imitate physiological glucose regulation (4). Unregulated open-source or Do-It-Yourself systems have been co-created by online communities and provide open-access algorithms for building one’s own AID system, allowing considerable user customization (5). Randomized controlled trials and real-world clinical studies examining the safety and efficacy of AID systems generally find a reduction in HbA_1c_ and hypoglycemia, as well as an increase of time in range (70-180 mg/dL or 3.9-10.0 mmol/L) to an average of 65-75%, even higher during nighttime and in select populations (1, 2, 6–9).

The objective of this paper is to provide an overview of the psychological implications of AID systems.

## Psychological Benefits

A growing number of trials and real-world observational studies focusing on AID systems have included person-reported outcome (PRO) measures to evaluate quality of life changes alongside glycemic benefits. Most of these report improvements in diabetes-specific distress or at least suggest technology advancements do not necessarily lead to added diabetes burden (10–15), although previous glycemic burdens might be exchanged for new technology burdens (15). While not found consistently, AID systems may also have positive effects on subjective and objective sleep of people with T1D (14, 16–21). Comparing two AID system generations in a randomized crossover trial among adolescents and adults, the advanced system improved satisfaction with the emotional and behavioral burden of glucose monitoring when compared with its predecessor; diabetes distress and hypoglycemia confidence were similar (22). Qualitative studies further detail the quality of life benefits of AID systems, describing a reduction of self-management burden and worries, increased flexibility and spontaneity, as well as improvements in relationships (23–25).

## Discontinuation

Despite potential glycemic and quality of life benefits, not all experiences are positive. While higher use of the algorithm has been associated with more optimal glycemic outcomes (9, 15, 26), consistent use of the closed-loop feature may drop soon, even to an average of 50% by six months in some – particularly younger - samples (15, 27). Furthermore, up to one-third of users of a first generation AID system stopped using the algorithm altogether by 3-6 months (15, 26, 28), although these numbers might be more hopeful for later technologies (7). In a demographically diverse adult population, 31% of AID users even never initiated the closed-loop feature (29). Reasons for discontinuation center on reimbursement and supply difficulties, technology frustrations, wear-related issues, unexpectedly high work load, fears and preferences, life intrusions, discouragement (e.g. when glycemic benefit expectations are not met), and other life stressors (24, 26, 28, 29). Preliminary quantitative studies focusing on a first-generation system suggest that higher baseline HbA_1c_ is predictive of lower use of the algorithm, potentially due to increased perceived self-care burden to keep the system going (28, 30), although those with high HbA_1c_ also stand more to gain (15, 31, 32). Early behavioral device data such as algorithm use and exits in the first 1-3 months may help to identify difficulties early on (30). Furthermore, from the sensor literature, it is known that individual perceptions of device benefits and burdens are key in continued device use (33). While benefits and burdens are relatable to most people, individuals may weigh the advantages and disadvantages of the system differently and this personal trade-off also deserves clinical attention (24).

In a mixed-method observational study, 5% of 874 people using or initiating a Do-It-Yourself closed-loop system self-identified as discontinuers in the year after the baseline assessment (34). Based on survey results, discontinuation was associated with older age and somewhat lower trust in the system, but not with other demographic, clinical or psychological factors (34). The most commonly stated reasons for discontinuation related to wanting to try different technologies and unmet benefit expectations (34). Qualitative themes described the mental burden associated with uptake/use, difficulties with adjusting settings, fear of disapproval by health professionals, technical or logistic barriers, and individual concerns (34).

## New Challenges

### Trust

In order to optimally benefit from AID, users have to release some control over diabetes management to the algorithm. This means developing an *appropriate* level over trust in the system, as technology to date is far from perfect and user vigilance is still needed (35). There often is an initial probation period of several weeks, in which users evaluate device accuracy by closely monitoring system actions and glucose levels, sometimes backed by a temporary increase of fingerpricks (23, 35). Trust may increase when glycemic results correspond more closely with personal beliefs about effect and safety (23, 35). At the same time, the algorithm’s learning process requires the user to refrain from micromanaging and let the system occasionally pick up falling or rising glucose levels at a slower and more dosed pace (23, 24). Limited possibilities for communicating day-to-day contextual variations may lead to additional frustrations (23, 35).

Even in experienced users, trust is highly context-specific, where people tend to have least confidence in the system’s proper handling of exercise and meal situations (35). Development of trust is also related to personal factors, with those having self-managed diabetes for many years reporting more skepticism (35). For some people confidence in the system is built only after a sense of understanding the algorithm (23), although for present commercial systems some of its workings may still feel like a black box and trust remains conditional on glucose levels in the here and now (24). Most people eventually find a collaborative partnership with the AID system to optimize glucose management and quality of life (23).

### Dependence and Deskilling

Many users of an AID system appreciate its ability to achieve glycemic outcomes beyond their own capabilities and to function as a back-up when needed, e.g. in case of unplanned physical activity, carbohydrate miscalculations or missed boluses (23, 36). For some, this is mixed with significant anticipatory anxiety about having to manage glucose on their own again (35), for example in case of system break-down. Firm reliance on the algorithm to address glucose fluctuations may also lead to forgetting to carry out key tasks, deskilling (e.g. in terms of carbohydrate counting) and less healthy eating (more snacking, increased portions, more high-fat energy-dense foods) (25, 36–39).

### Compensatory Behaviors

Behaviors that contribute to optimal glycemic outcomes in open loop may bring new challenges in the context of closed loop, requiring significant cognitive and emotional efforts to give over enough control to an – as yet – imperfect system in order for it to improve performance (24). Frustrations with and distrust in the proper functioning of the AID system may lead to several user actions to retain personal control. Efforts to override or trick the system into delivering extra insulin may be more common than realized, as many people are hesitant to tell health care providers and peers about these actions (24). Compensatory behaviors include temporarily stepping back to open loop or employing workarounds such as entering fake carbohydrates (also called phantomboluses) (24, 35). Furthermore, as the algorithm works best with few external challenges, some people actively limit physical activity or intake of carbohydrates to further increase their time in range (24, 37, 40). Given known technology shortcomings, having an open dialogue about the goals and consequences of these compensatory behaviors is the most constructive way for optimizing human-device interactions (24).

### Bodily Concerns

Bodily concerns are important reasons for diabetes technology non-adoption or discontinuation in general (41, 42). These range from practical frustrations as well as pain and discomfort to more aesthetic and experiential aspects, such as increased self-consciousness, unwanted social visibility, and altered body or self-image (43–45).

Current AID systems may echo (36) as well as complicate these issues by requiring people to wear two or sometimes even three devices on the body. This may particularly become apparent in the context of relationship intimacy, where people simultaneously manage prevention of device dislodgements and not hurting a partner as well as their relationship itself (46).

## Developments and Future Outlook

### Diversity Perspective

An important limitation of the studies reviewed in this paper is that they have mostly included majority populations without significant health disparities. It remains to be determined whether their challenges are generalizable to underrepresented and underserved populations. Specific challenges may go beyond barriers to technology use related to costs, availability and prescription bias. For example, in a small study among 32 adults with T1D treated at an academic urban safety-net hospital who were prescribed a first generation AID system, black and Hispanic people were overrepresented in the group who never initiated auto-mode despite similar insurance and educational level to the rest of the sample (29). Given potential glycemic and quality of life benefits, future research is encouraged to identify strategies for increasing uptake and continued use of AID systems in underserved populations (29).

A broader age perspective is also needed. While some device struggles appear to be similar across the lifespan, others may differ in expression and emphasis for different age groups (47). For example, adolescents and young adults may struggle with AID systems due to interference with regular developmental tasks related to body image, identity, independence and peer relations (27, 42) while older adults may face additional management challenges including a higher risk of severe hypoglycemia and long-term complications as well as sleep disruption and problems related to cognition, dexterity, and vision (19, 48).

### Measurement of Psychological Impact

The impact of AID systems may go well beyond glycemic parameters and meaningfully influence quality of life. Regular person-reported outcome (PRO) assessment may be of value, e.g. to track psychological problems interfering with optimal AID functioning such as fear of hypoglycemia and related behaviors including taking many extra carbohydrates at night. However, in a preliminary study, common measures of diabetes distress and worries about hypoglycemia did not predict algorithm use after one year; therefore, these tools might not tap sufficiently into the psychology of technology use (30). Instruments are available to measure specific perceptions and experiences related to AID therapy, including the INSPIRE questionnaires and adaptations of the Technology Acceptance Scale (10, 49). In the upcoming years, these will need to be updated according to new psychological issues arising with further technology advancements.

### Psychological Factors as Selection Criteria

The assessment of characteristics in light of identifying predictors of device success is not straightforward. Behavioral and medical factors such as ≥4 blood glucose checks per day and higher sensor use prior to algorithm initialization have been associated with more frequent use of the algorithm and higher improvements in glycemic outcomes (26, 27, 50), but there is a limited number of studies and results are not always consistent, e.g. with respect to HbA_1c_ level. Furthermore, personal definitions of benefit may differ (e.g. in terms of glucometrics, acute complications, continued system use, person-reported outcomes) and getting the support from health professionals in transitioning to AID technology may itself stimulate increased diabetes self-care engagement (30, 38, 51). Selection procedures may even be counterproductive for the working relation between the person with T1D and their health professionals in terms of inappropriate subjective gatekeeping to technology access (38, 52). In the REPOSE trial, staff described applying their own perceptions of personal and psychological suitability (e.g. in terms of higher education, technological comprehension) in recommending people for insulin pump therapy in regular care, which proved to be incorrect in multiple instances with random therapy allocation (51). Similar assumptions were found and challenged in the CLOuD trial focusing on AID technology (38). In the context of AID therapy, people with suboptimal self-management behaviors and glycemic outcomes at system start described relatively easy adaptation and great benefits, while relinquishing control to the algorithm was especially challenging for people with lower initial HbA_1c_ and higher personal standards for diabetes management (24). In this respect, measurement of psychological factors – similar to social and health factors such as socioeconomic status, social support, visual or dexterity impairments, psychopathology, cognition - should only serve as input for stimulating an open discussion about AID initiation/continuation and mapping the support needed for an individual to access optimal benefits of advanced technologies.

### Integration of Psychological Information in AID Algorithms

Integration of other information to algorithms may further increase AID performance (1). One factor to consider is the effect of psychological stress on insulin sensitivity and glucose levels. While there is large inter- and intra-individual variability in stress-reactivity, daily stressors may increase glucose variability (53, 54). Therefore, more research is warranted to examine the potential predictive contribution of stress and other situational/behavioral factors to AID algorithms (54). To capture the complexity of the stress – glucose link in the context of AID therapy, these studies preferably incorporate ecological momentary assessments over longer time periods (54).

### Psychological Counseling and Support

“One size fits all” does not apply to T1D care (55), with some people making well-informed and well-considered personal decisions against AID adoption and continued use. However, many people currently unnecessarily do not fully benefit from AID therapy. Apart from changes at the policy level (e.g. broader reimbursement), health professionals as well as peers have an important role to play in this respect. This starts with increased awareness of their own technology attitudes and experiences (38, 51). Opportunities for support further include guiding appropriate expectations, offering structured education programs and providing tailored strategies for managing device hassles as personal preferences may differ (28, 35, 52, 56, 57). More studies are needed to assist the development of more concrete conversational and interventional tools in this respect. Interesting developments include behavioral telehealth interventions such as ONBOARD (focusing on sensor use, including the themes of discomfort, data overload, trust and unwanted social attention) and virtual reality exposure to technology barriers relating to body image, hassles of use, worries about losing skills, and unwanted social attention (58, 59).

## Conclusion

AID systems offer the potential of significant glycemic and quality of life benefits to people with T1D. As long as devices remain visibly worn on the body and still require at least some human effort, a better understanding of person-technology interactions remains key. Psychology offers several tools for measuring the quality of life impact of AID systems and may bring important insights for addressing cognitive, emotional or behavioral barriers towards optimal use. Central to all efforts is an ongoing dialogue, with efforts to maximize benefits and minimize burdens of AID therapy and with sensitivity to the personal trade-off between both.

## Author Contributions

The author confirms being the sole contributor of this work and has approved it for publication.

## Conflict of Interest

The author declares that the research was conducted in the absence of any commercial or financial relationships that could be construed as a potential conflict of interest.

## Publisher’s Note

All claims expressed in this article are solely those of the authors and do not necessarily represent those of their affiliated organizations, or those of the publisher, the editors and the reviewers. Any product that may be evaluated in this article, or claim that may be made by its manufacturer, is not guaranteed or endorsed by the publisher.
